# DronePropA: Motion trajectories dataset for defective drones

**DOI:** 10.1016/j.dib.2025.111589

**Published:** 2025-05-01

**Authors:** Mohamed A.A. Ismail, Mohssen E. Elshaar, Ayman Abdallah, Quan Quan

**Affiliations:** a Interdisciplinary Research Center for Aviation and Space Exploration, King Fahd University of Petroleum & Minerals (KFUPM), Dhahran 31261, Saudi Arabia; b Aerospace Engineering Department, King Fahd University of Petroleum & Minerals (KFUPM), Dhahran 31261, Saudi Arabia; c School of Automation Science and Electrical Engineering, Beihang University, Beijing 100191, PR China

**Keywords:** Health monitoring, Multicopter drone, Propeller cracks, Fault detection, Unbalance

## Abstract

Unmanned aerial vehicles, or drones, are increasingly deployed in critical applications that demand exceptional safety and reliability. However, as drones become integral to industries such as logistics, agriculture, and public safety, reliability issues with core components like propellers can lead to serious safety risks and financial losses. Propellers typically have high failure rates, especially in harsh conditions, which has encouraged research into effective health monitoring techniques for early fault detection. Despite these efforts, existing datasets on faulty propellers remain limited in scale, diversity, and coverage of fault types and severity levels. This data article introduces a comprehensive dataset composed of motion trajectories and flight logs of 130 flight sequences for a commercial quadcopter platform. This dataset includes different flight paths, fault types, and severity levels. The dataset primarily comprises onboard sensor readings from the drone and the corresponding mission trajectory logs. All faults develop while the drone operates normally, with no significant impact on performance across flight phases. These faults include minor cracks, edge damage, and holes, which are some of the popular propeller failures. This dataset is valuable for exploring effective fault detection methods and predictive maintenance capabilities.

Specifications Table

**Table:** 

Subject	Aerospace Engineering.
Specific subject area	*Health Monitoring for Unmanned Aerial Vehicles.*
Type of data	Dataset in “.mat” format, Figures, Table
Data collection	*The dataset contains 130 flight data sequences for healthy and faulty propellers in five trajectories. The faulty propellers involve three fault types for all trajectories: an edge cut, a propeller crack, and a surface cut. In addition, there are three fault severity levels for each type. The flight data sequences are conducted on three drones. Each flight data sequence involves drone position, velocity, orientation angles, thrust commands, and battery levels, recorded via onboard Inertial moment units (IMUs).*
Data source location	*Interdisciplinary Research Center for Aviation and Space Exploration, King Fahd University of Petroleum & Minerals (KFUPM), Dhahran 31,261, Saudi Arabia.*
Data accessibility	Repository name: DronePropA: Motion Trajectories Dataset for Commercial Drones with Defective Propellers (Mendeley Data) Data identification number: 10.17632/ftdyxrr3c5 Direct URL to data: https://doi.org/10.17632/ftdyxrr3c5
Related research article	*M. E. Elshaar, M.A.A. Ismail, A. Abdallah, Q. Quan, Fault Diagnosis of Drone Propellers using Inner Loop Dynamics, ICCAD’25: 9th International Conference on Control, Automation and Diagnosis, 2025 July 1–3, Barcelona, Spain.*

## 1 Value of the Data

• **Support for Fault Detection and Predictive Maintenance:** Labeled fault types and severity levels can be utilized to develop machine learning (ML) models that classify drones as either healthy or faulty. The residuals from these ML models can be monitored during flight to facilitate potential emergency landings or tracked offline as a measure for predictive maintenance and repair.
• **Support for System Identification and Fault-Tolerant Control:** This data can be employed to identify a dynamic model for a quadcopter drone. The model parameters can represent different faults, such as the mechanical efficiency of the propeller. Utilizing this dataset and the associated fault conditions, a fault-tolerant control system can be explored.
• **Comprehensive Fault Coverage and High-Sampling**: The dataset includes flight trajectories and sensor logs recorded under various propeller fault types (propeller cracks, edge damage, surface cuts) and severity levels, providing a wide range of scenarios for analysis. These fault types are selected to match most cited propeller failures in literature. Data from multiple onboard sensors are measured at sampling frequency of 1 kHz, ensuring consistent and high-resolution time-series data for covering faults along a wide bandwidth.

## 2 Background

Recent research has focused on real-time health monitoring of drones to enable early detection of potential issues and ensure safe operation [1,2]. However, available datasets on drone flight dynamics under faulty conditions are limited in both scope and diversity. This data article presents a comprehensive dataset [3] collected from 130 flight experiments, encompassing a number of similar drones, fault types, severities, and flight trajectories. Edge cuts, cracks, and unbalance are critical propeller faults that significantly impact the performance, safety, and reliability of UAVs. Edge cuts occur when the leading or trailing edges of the blades sustain damage due to high aerodynamic forces concentrated in these regions [4]. Often caused by environmental debris or aerodynamic stresses, edge cuts disrupt airflow, increase drag, and reduce thrust efficiency, directly affecting flight stability [5]. Cracks are fractures that weaken the propeller's structural integrity [6]. These can result from fatigue, impact damage, or material inconsistencies, compromising both structural strength and aerodynamic performance. Cracks lead to increased vibrations, which can escalate into catastrophic failure if left unaddressed [7]. Unbalance arises from mass asymmetry due to damage, repairs, or material loss, leading to centrifugal forces that induce vibrations. This unbalance degrades sensor accuracy and affects flight control, posing significant challenges to UAV operation and stability [8].

The dataset captures detailed sensor logs of drone states, including position, velocity, orientation, thrust commands, and battery levels, recorded via onboard Inertial moment units (IMUs) and additional flight monitors. This rich set of in-flight data allows for a comprehensive analysis of how various faults and severities affect flight dynamics and overall performance across different manoeuvres and speeds. The primary goal of this dataset is to provide insights into fault-induced changes in flight behaviour, establishing a valuable resource for the development and benchmarking of advanced fault detection and health monitoring algorithms for drones.

## 3 Data Description

**Fig. 1:**
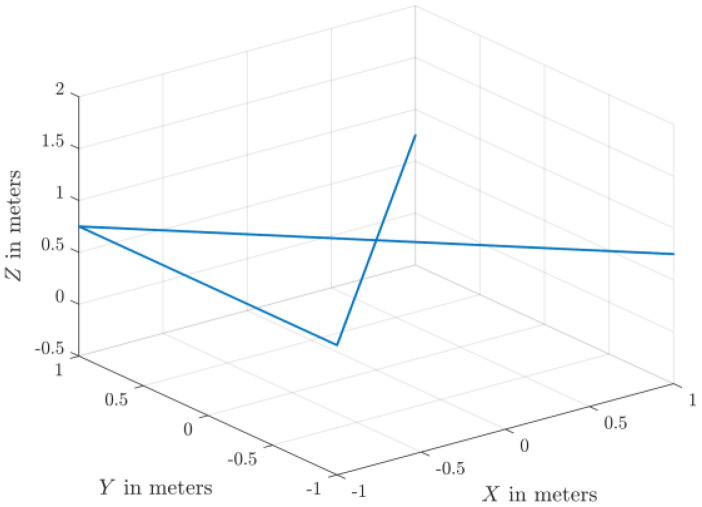
Diagonal motion trajectory.

**Fig. 2:**
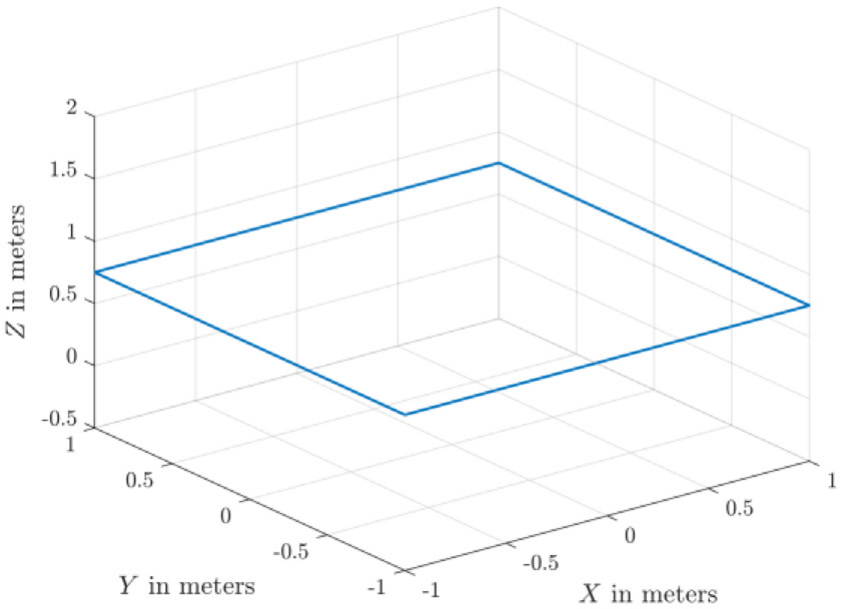
Square-shaped trajectory.

**Fig. 3:**
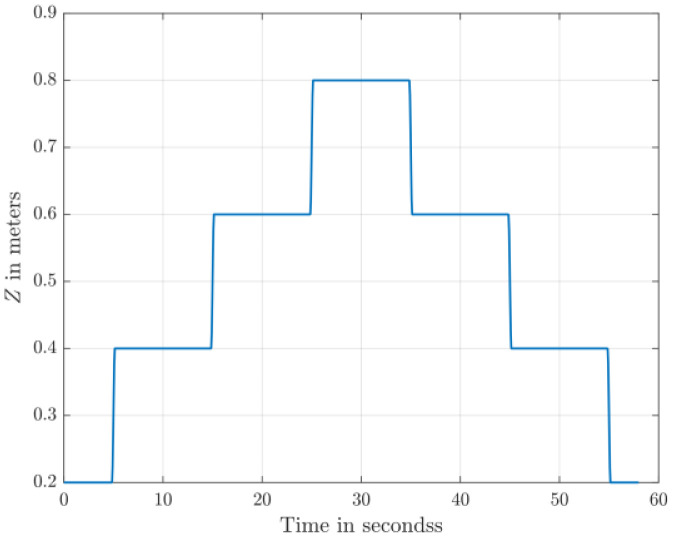
Ascending and descending in steps trajectory.

**Fig. 4:**
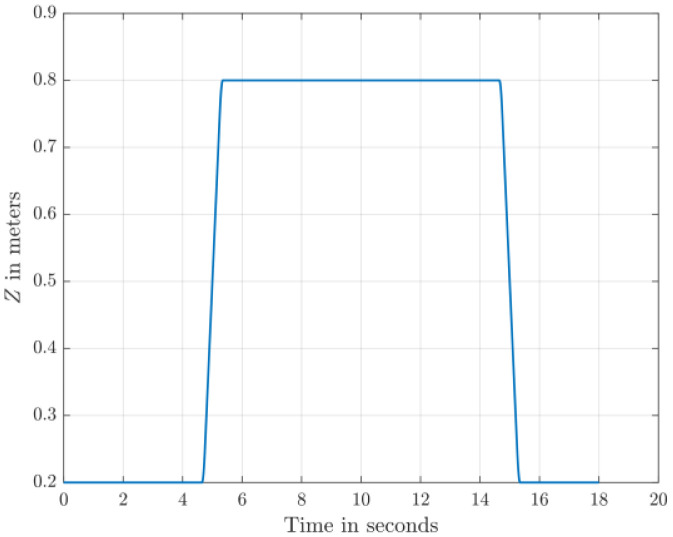
Ascending and descending in one shot.

**Fig. 5:**
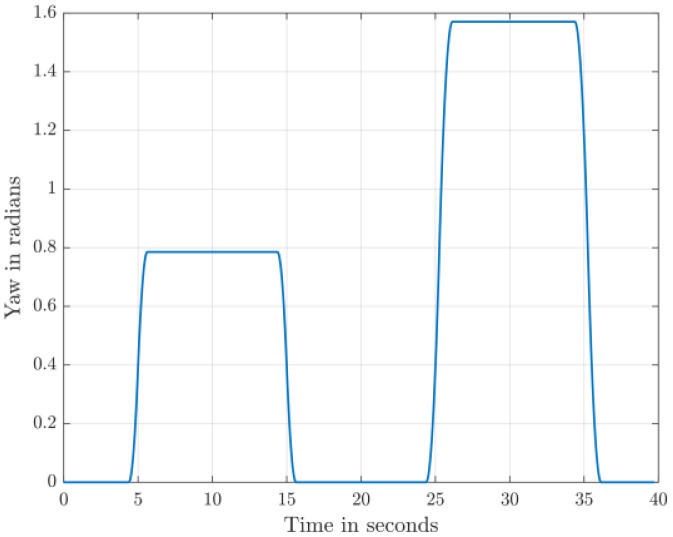
Yawing maneuver.

**Fig. 6:**
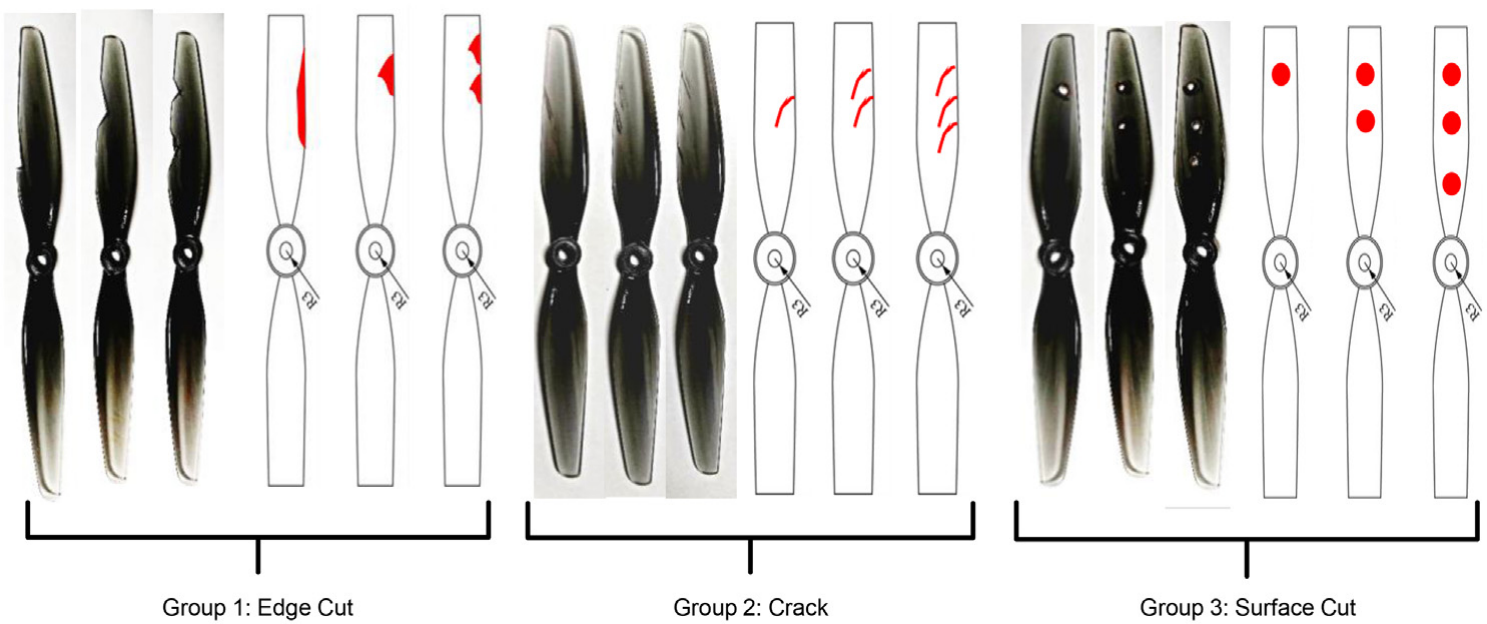
Photographs of the three fault groups for the drone propeller.

The dataset comprises 130 files detailing healthy and faulty propellers in five trajectories, and they are available in [3]. They were used for developing model-based fault diagnosis for drone propeller in previous research [9]. To reduce measurement uncertainties from a single setup, the healthy data are collected from three distinct drones of the same type. Across all trajectories, faulty propellers exhibit one of three fault types: an edge cut, a crack, or a surface cut. Each fault type is represented at three severity levels and tested at two distinct speeds. The drone follows five trajectories (Fig. 1, Fig. 2, Fig. 3, Fig. 4, Fig. 5): a cross pattern where it moves along the diagonals of a 1 × 1 m square; a square path along the edges of a 1 × 1 m square; an ascent in 0.2 m increments from 0.2 m to 0.8 m; a direct climb from 0.2 m to 0.8 m; and a yawing sequence, rotating by 45, −45, 90, and −90 degrees. Each trajectory is experimented 26 times. The main experiments focus on a single drone, covering all 9 fault types and severity levels combinations, along with a healthy condition, at two speeds: (0.333 m/s, 0.52 rad/s) and (2 m/s, 3.14 rad/s) . Each healthy scenario is repeated three times, while two additional healthy drones of the same model complete all trajectories at the maximum speed (2 m/s, 3.14 rad/s) under healthy conditions for reference. All system measurements are sampled at 1 kHz. As shown in Fig. 6, faults are classified into three groups: edge cuts, cracks, and surface cuts, which represent common propeller failures. Each fault group is represented by three severity levels, achieved by enlarging the faulty area in Group 1 or replicating the fault in Groups 2 and 3. The propeller model used is the HQ Durable 7 × 4.5 [10].

The dataset contains a total of 130 MATLAB “.mat” files, amounting to 4.22 GB in size, with individual file sizes ranging from 20 to 45 MB. All MATLAB “.mat” files contain three consistent variables: commander_data, QDrone_data, and stabilizer_data. Each variable is a matrix where rows represent time-series signals recorded during the experiments. Full MATLAB codes for displaying the data parameters and features are available in [11]. A detailed description of these variables is provided in Table 1.

**Table 1:** Description of the variables in MATLAB “.mat” data file.

Matlab-Quanser Variable Name	Parameter	Row Index	Unit & Explanation
Commander_data	Time	1	Time data
	Measured position X	22	In meters, from the reference trajectory
	Measured position Y	23	In meters, from the reference trajectory
	Measured position Z	24	In meters, from the reference trajectory
	Measured angle Yaw	25	In radians, from the reference trajectory
	Ref position X	26	In meters, from the Optitrack system
	Ref position Y	27	In meters, from the Optitrack system
	Ref position Z	28	In meters, from the Optitrack system
	Ref angle Yaw	29	In radians, from the Optitrack system
	Ref Thrust	34	In newtons, an internal variable from the drone controller.
	Ref Roll Angle	35	In radians, an internal variable from the drone controller.
	Ref Pitch Angle	36	In radians, an internal variable from the drone controller.
	Ref Yaw Rate	37	In rad/s, an internal variable from the drone controller.
QDrone_data	Time	1	Time data
	Measured Roll	2	In radians, from the IMU unit #1
	Measured Pitch	3	In radians, from the IMU unit #1
	Measured Yaw	4	In radians, from the IMU unit #1
	Measured Roll Rate	5	In rad/s, from the IMU unit #1
	Measured Pitch Rate	6	In rad/s, from the IMU unit #1
	Measured Yaw Rate	7	In rad/s, from the IMU unit #1
	Measured Roll Acceleration	8	In rad/s^2^, from the IMU unit #1
	Measured Pitch Acceleration	9	In rad/s^2^, from the IMU unit #1
	Measured Yaw Acceleration	10	In rad/s^2^, from the IMU unit #1
	Measured Roll	11	In radians, from the IMU unit #2
	Measured Pitch	12	In radians, from the IMU unit #2
	Measured Yaw	13	In radians, from the IMU unit #2
	Measured Roll Rate	14	In rad/s, from the IMU unit #2
	Measured Pitch Rate	15	In rad/s, from the IMU unit #2
	Measured Yaw Rate	16	In rad/s, from the IMU unit #2
	Measured Roll Acceleration	17	In rad/s^2^, from the IMU unit #2
	Measured Pitch Acceleration	18	In rad/s^2^, from the IMU unit #2
	Measured Yaw Acceleration	19	In rad/s^2^, from the IMU unit #2
	Battery Level	24	In volts
	Measured Roll Rate	27	In rad/s, from the Gyroscope unit #1
	Measured Pitch Rate	28	In rad/s, from the Gyroscope unit #1
	Measured Yaw Rate	29	In rad/s, from the Gyroscope unit #1
	Measured Acceleration along X	30	In m/s^2^, from the Accelerometer unit #1
	Measured Acceleration along Y	31	In m/s^2^, from the Accelerometer unit #1
	Measured Acceleration along Z	32	In m/s^2^, from the Accelerometer unit #1
	Measured Roll Rate	33	In rad/s, from the Gyroscope unit #2
	Measured Pitch Rate	34	In rad/s, from the Gyroscope unit #2
	Measured Yaw Rate	35	In rad/s, from the Gyroscope unit #2
	Measured Acceleration along X	36	In m/s^2^, from the Accelerometer unit #2
	Measured Acceleration along Y	37	In m/s^2^, from the Accelerometer unit #2
	Measured Acceleration along Z	38	In m/s^2^, from the Accelerometer unit #2
	Height Range Data	46	In meters, from the height sensor
	Front Left Motor Command	47	In volt percentage
	Front Left ESC Command	48	In volt percentage
	Front Right Motor Command	49	In volt percentage
	Front Right ESC Command	50	In volt percentage
	Back Left Motor Command	51	In volt percentage
	Back Left ESC Command	52	In volt percentage
	Back Right Motor Command	53	In volt percentage
	Back Right ESC Command	54	In volt percentage
Stabilizer_data	Time	1	Time data
	Flight Mode	7	Logs the current flight mode of the drone.

The descriptions of the data file names according to the health and the operating conditions are arranged as follows in Table 2.

**Table 2:** Data files naming rules.

Code	Possible values
F	{1,2,3}, where F#n: Fault group #n
SV	{1,2,3}, where SV#n: Severity level #n
SP	{1,2}, where SP#n: Speed #n SP1: Maximum Speed of (2 m/s, 3.14 rad/s) SP2: Maximum Speed of (0.333 m/s, 0.52 rad/s)
t	{1,2,3,4,5}, where t#n: Trajectory #n t1: Diagonal motion (The drone moves back and forth along the diagonals of a 1 × 1 m square) t2: Square-shaped motion (The drone moves along the edges of 1 × 1 m Square) t3: Ascending and descending in steps (The drone altitude increases from 0.2 m to 0.8 m in 0.2 m Increments) t4: Ascending and descending in one shot (The drone altitude increases from 0.2 m to 0.8 m directly) t5: Yawing maneuver (The drone rotates by 45°, −45°, 90°, and −90° independently)
D	{1,2,3}, where D#n: Drone #n Healthy trajectories are generated for three drones, while faulty trajectories are generated for drone 1 only.
R	{1,2,3}, where R#n: Repetition #n When the same combination of the codes other than “R” is repeated more than once (The same experimet is repeated).

*Examples:*

1. F0_SV0_SP1_t1_D1_R1.mat: a flight log dataset for a fully healthy drone number 1 with a Maximum Speed of (2 m/s, 3.14 rad/s) along Trajectory 1.
2. F3_SV1_SP2_t1.mat: a flight log dataset for a faulty propeller drone of Fault Type 3, Severity Level 1 with a Maximum Speed of (0.333 m/s, 0.52 rad/s) along Trajectory 1.
3. F1_SV3_SP1_t3.mat: a flight log dataset for a faulty propeller drone of Fault Type 1, Severity Level 3 with a Maximum Speed of (2 m/s, 3.14 rad/s) along Trajectory 3.

## 4 Experimental Design, Materials and Methods

**Fig. 7:**
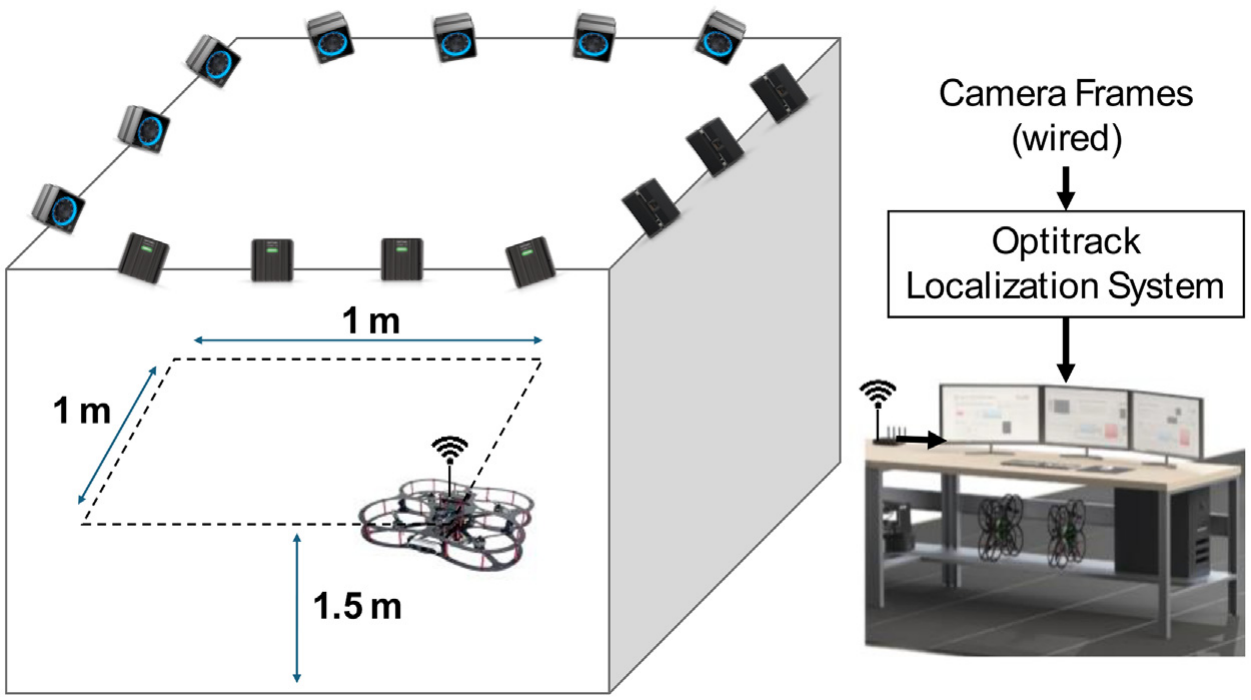
Schematic of Drone Test Setup based on Quanser™ AVRS.

The test setup (Fig. 7) is based on Quanser™ AVRS (Autonomous Vehicles Research Studio) platform for testing and developing aerial robots [12]. It comprises multiple quadcopters, a ground control station based on MATLAB and Optitrack™ vision-based positioning system of infrared cameras and optical markers. Optitrack™ system needs a special calibration software and procedures that are described in [12]. Based on 14 cameras, the Optitrack™ calibration achieves a positioning accuracy of ±3 mm for the drone position along X, Y and Z axes. A drone model Quanser™ Qdrone 2 [13] is used with a maximum takeoff weight of 1.5 Kg and a hovering time of 8 min. The drone involves 2 IMU units (model IIM-42652), an onboard data acquisition card, and a bunch of onboard digital & analog I/O channels for motor and esc monitors. The drone position is continuously measured through the Optitrack™. All drones in this research are new and their onboard sensors were calibrated by the drone manufacturer. In addition, the flight test data for the healthy drones indicate smooth motion trajectories and they are ready for fault-injection research by replacing a defective propeller. All data are recorded at sampling frequency of 1 kHz. The motion trajectories (Figs 1–5) are written in MATLAB in the ground control station, and they are transferred to the drone using Wi-Fi communication channel.

## Limitations

This dataset was collected under controlled indoor conditions, meaning it does not consider environmental factors such as wind, turbulence, or temperature variations, which could affect how faults impact drone dynamics. Additionally, the faults were artificially introduced to the propellers. There may be a mismatch between artificial faults and service-induced faults.

## Ethics Statement

This work does not involve human subjects, animal experiments, or any data collected from social media platforms. The authors have read and follow the ethical requirements for publication in Data in Brief.

## CRediT Author Statement

**Mohamed AA Ismail**: Design of experiments, Reviewing and Original draft co-writing. **Mohssen Elshaar**: Conduct of experiments and Original draft co-writing. **Ayman Abdallah**: Reviewing, Supervision and Editing. **Quan:** Reviewing and Supervision.

## Data Availability

Mendeley DataDronePropA: Motion Trajectories Dataset for Commercial Drones with Defective Propellers (Original data)
